# Supplementary material for: Directed evolution of prenylated FMN-dependent Fdc supports efficient in vivo isobutene production
Source: Nat Commun. 2021 Sep 6;12:5300. doi: 10.1038/s41467-021-25598-0 (PMC8421414; doi:10.1038/s41467-021-25598-0)
Supplement: Supplementary file 1 — Supplementary Information file [file 41467_2021_25598_MOESM1_ESM.pdf]

Directed evolution of prenylated FMN-dependent Fdc supports efficient *in vivo* isobutene production

Saaret *et al*.

Supplementary Table 1. **Initial *in vivo* screening of Fdc variants for wild-type 3-methylcrotonic acid decarboxylation activity.**

pETDuet	Fdc Uniprot code	Isobutene (ppm)	
		Average	Std Dev
CiFdc-UbiX	A0A0D2AQI6	16.4	0.8
SmFdc-UbiX	M3DF95	5.9	0.7
PrFdc-UbiX	W6QKP7	9.2	1.5
ApFdc-UbiX	A0A0F0IHE5	108.9	8.4
NfFdc-UbiX	A1DCG7	108.0	7.4
CcFdc-UbiX	W9YNA8	125.2	9.2
PcFdc-UbiX	A0A0G4P429	19.8	2.9
AnFdc-UbiX	A2QHE5	13.2	3.1
BfFdc-UbiX	M7THT1	7.8	1.1
PpFdc-UbiX	A0A094IED9	8.8	2.4
CsFdc-UbiX	A0A0D2DPQ1	13.4	1.1
CpFdc-UbiX	W9WWR1	18.0	2.2
CdFdc-UbiX	B9WJ66	4.8	1.3
ScFdc-UbiX	Q03034	17.6	1.3
TaFdc-UbiX	G9NLP8	330.0	29.1
UbiX alone	N/A	3.3	1.4
empty plasmid	N/A	3.2	1.2

Note: The Fdc was co-expressed with UbiX in a pETDuet plasmid with Fdc in MCS1 (multiple cloning site 1) with N-terminal 6His-tag and UbiX in MCS2.

Supplementary Table 2. Calculation of DFT optimised zero-point energy (ZPE) corrected energies for isobutene release from 3-methylcrotonic acid Int3.

3-methylcrotonic acid	Single point energy		ZPE correction (Hartrees)	Single point + ZPE correction	
	Hartrees	kJ/mol		Hartrees	kJ/mol
Int3	-8392.956148	0.00	3.186926	-8389.769222	0.00
TS	-8392.925221	81.20	3.181997	-8389.743224	68.26
isobutene	-8392.943099	34.26	3.183405	-8389.759694	25.02

Supplementary Table 3. Calculation of DFT optimized zero-point energy (ZPE) corrected energies from DFT for propene release from crotonic acid Int3.

Crotonic acid	Single point energy		ZPE correction (Hartrees)	Single point + ZPE correction	
	**Hartrees**	**kJ/mol**		**Hartrees**	**kJ/mol**
Int3	-8353.620958	0.00	3.159167	-8350.461791	0.00
TS	-8353.583364	98.70	3.154838	-8350.428526	87.34
Propene	-8353.603756	45.16	3.154652	-8350.449104	33.31

Supplementary Table 4. Natural charge analysis for DFT models with 3-methylcrotonic acid.

Moiety	Summed natural charges				
	Int3	TS	Product	Prod - Int3	TS - Int3
substrate (3-methylcrotonic acid)	0.11	0.26	-0.02	-0.13	0.15
Cα	-0.47	-0.53	-0.49	-0.02	-0.06
Cβ	-0.03	0.15	-0.05	-0.02	0.18
C4a	0.01	-0.07	-0.05	-0.06	-0.09
N5	-0.46	-0.36	-0.30	0.16	0.11
C1'	-0.04	0.08	0.15	0.19	0.12
prFMN	0.01	0.05	0.05	0.05	0.05
isoalloxazine	-0.62	-0.89	-0.69	-0.07	-0.27
additional prenyl carbons	0.25	0.43	0.51	0.26	0.18
‘tail’	0.26	0.25	0.26	-0.01	-0.01
Cɣ	-0.69	-0.72	-0.69	0.00	-0.03
Cɣ'	-0.70	-0.73	-0.70	0.00	-0.03

Supplementary Table 5. Natural charge analysis for DFT models with crotonic acid.

Moiety	Summed natural charges				
	Int3	TS	Product	Prod - Int3	TS - Int3
substrate (crotonic acid)	0.12	0.21	-0.02	-0.14	0.09
Cα	-0.47	-0.52	-0.49	-0.02	-0.05
Cβ	-0.23	-0.10	-0.24	-0.01	0.14
C4a	0.01	-0.06	-0.06	-0.06	-0.06
N5	-0.47	-0.35	-0.29	0.18	0.12
C1'	-0.04	0.06	0.17	0.21	0.11
prFMN	0.03	0.05	0.06	0.03	0.02
isoalloxazine	-0.62	-0.83	-0.70	-0.08	-0.21
additional prenyl carbons	0.26	0.42	0.53	0.27	0.16
‘tail’	0.27	0.25	0.25	-0.01	-0.01
Cɣ	-0.69	-0.72	-0.71	-0.02	-0.03
H	0.26	0.24	0.23	-0.03	-0.02

Supplementary Table 6. Primers for mutagenesis using the Q5® Site-Directed Mutagenesis Kit (New England Biolabs).

Mutation	Primers (5’->3’)
T395M to *An*Fdc to yield *An*FdcI	GGCGGGTTATATGATTCACCGCC<br>TTATGATTGAAAACCACATCG
R435P and P438W to *An*FdcI to yield *An*FdcII	GCCCGGCTTCTGGCTGATTCCGTACATGGGTC<br>ACGTCTTCAAACAGCGTTTCATCCATACCCGG
T405M to *Ta*Fdc to yield *Ta*FdcI	ACCGGGTTTTATGATTCATCGTCTG<br>TTCTGGGTAAACACAACATC
F404Y, T405M, V445P and Q448W to *Ta*Fdc to yield TaFdcII	First (for F404Y and T405M)<br>AAACCGGGTTATATGATTCATCGTCTGATTC<br>CTGGGTAAACACAACATC<br>Followed by (V445P and Q448W)<br>TTTTGGCTGATCCCGTATATGAG<br>GCCCGGAACATCATCAAAAAAAACTTCATC

Supplementary Table 7. Crystallographic data and refinement statistics.

	***Ta*Fdc**	***Ta*FdcV**	***Ta*FdcV-2-butynoic**	***Ta*FdcV-crotonic**	***An*FdcI**	***An*FdcII**
PDB code	7NEY [https://doi.org/10.2210/pdb7NEY/pdb]	7NF0 [https://doi.org/10.2210/pdb7NF0/pdb]	7NF1 [https://doi.org/10.2210/pdb7NF1/pdb]	7NF2 [https://doi.org/10.2210/pdb7NF1/pdb]	7NF3 [https://doi.org/10.2210/pdb7NF3/pdb]	7NF4 [https://doi.org/10.2210/pdb7NF4/pdb]
Organism	*Trichoderma atroviride*	*Trichoderma atroviride*	*Trichoderma atroviride*	*Trichoderma atroviride*	*Aspergillus niger*	*Aspergillus niger*
Resolution range (Å)	63.19 – 1.74 (1.785 – 1.74)	63.50 – 1.35 (1.385 – 1.35)	64.65 – 1. 77 (1.80 – 1.77)	115.12 – 1.33	42.09 – 1.10 (1.12 – 1.10)	85.07 – 1.69 (1.72 – 1.69)
Wavelength (Å)	0.9686	0.9795	0.9686	0.9159	0.9763	0.9763
Space group	P 3_2 2 1	P 3_2 2 1	P 3_2 2 1	P 3_2 2 1	P21 21 2	P21 21 2
Cell dimensions *a*, *b*, *c* (Å)	74.212, 74.212, 346.561	74.593, 74.593, 345.772	74.647, 74.647, 345.752	74.540, 74.540, 345.360	95.910, 63.850, 87.820	64.450, 97.010, 177.030
Mean I/sig(I)	12.6 (2.3)	10.4 (1.2)	6.8 (2.2)	6.7 (1.2)	15.7 (2.2)	7.8 (1.0)
Completeness (%)	99.98 (99.7)	99.7 (93.7)	99.93 (98.7)	99.89 (99.4)	97.89 (82.7)	99.85 (100)
Multiplicity	26.4 (12.5)	8.8 (4.3)	9.1 (5.9)	9.2 (6.9)	4.9 (2.7)	5.4 (5.5)
Total reflections	3044619 (70794)	2149829 (48807)	1006477 (31735)	2362949 (85892)	1043008 (24262)	677172 (33946)
Unique reflections	115391 (5671)	245351 (11383)	110826 (5368)	256349 (12523)	213550 (8943)	124712 (6130)
R_{meas}	0.185 (0.833)	0.103 (1.346)	0.226 (0.798)	0.165 (1.459)	0.059 (0.849)	0.161 (1.704)
R_{work}	0.182	0.166	0.175	0.162	0.158	0.191
R_{free}	0.215	0.184	0.207	0.181	0.172	0.220
No. non-hydrogen atoms	8124	8678	8321	8989	4646	8332
Average B-factor (Å^2)	25.350	15.618	22.445	16.037	11.888	21.828
RMS bond angles (°)	1.731	1.923	1.718	1.825	2.019	1.663
RMS bond lengths (Å)	0.011	0.014	0.011	0.014	0.018	0.011

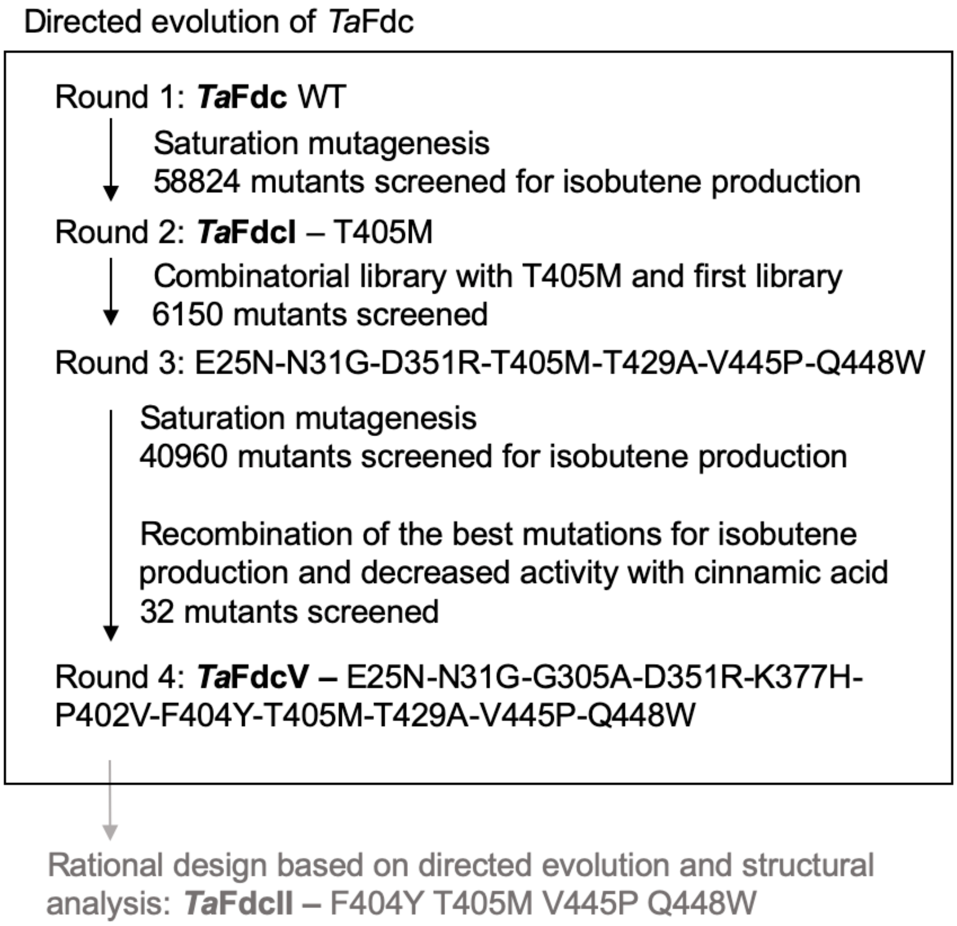

Supplementary Figure 1. The directed evolution of *Ta*Fdc wild-type to *Ta*FdcV with superior isobutene production and the following rational design of *Ta*FdcII.

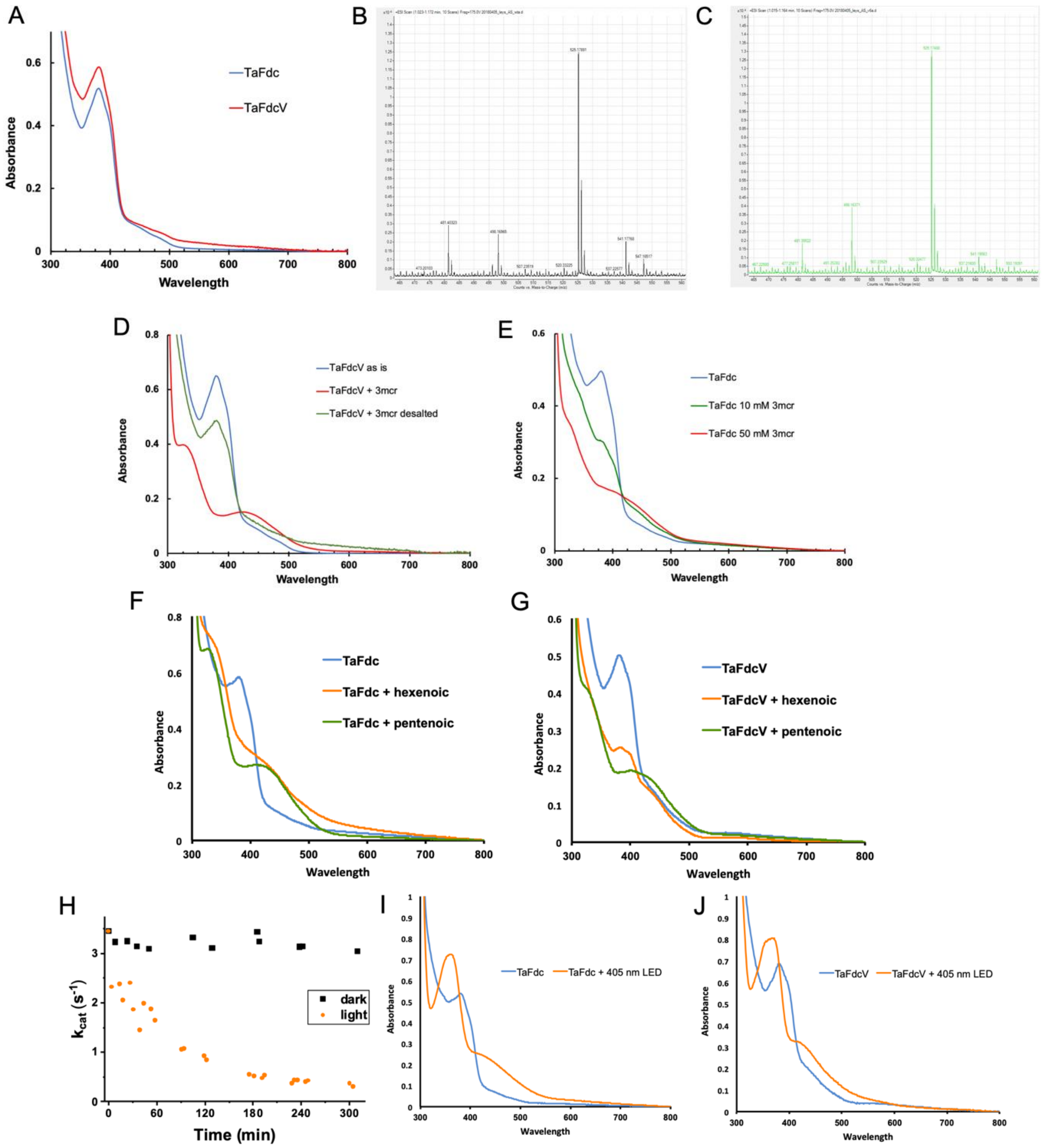

Supplementary Figure 2. Solution data for *Ta*Fdc and *Ta*FdcV. (A) UV-Vis spectra of purified *Ta*Fdc (blue) and *Ta*FdcV (red) with a sharp peak at 380 nm. (B, C) ESI-MS spectrum of *Ta*Fdc (B) and *Ta*FdcV (C) confirming the presence of prFMN in iminium form ($[M+H]^+ = 525.17$). (D) Splitting of the UV-Vis spectrum of *Ta*FdcV upon incubation with 10 mM 3-methylcrotonic acid (3mcr) to peaks at 325 nm and 430 nm (red); desalting of *Ta*FdcV incubated with 3mcr recovers the 380 nm $prFMN^{iminium}$ peak (green). (E) Incubation of *Ta*Fdc with 10 mM 3-methylcrotonic leads to a partly split spectrum (green) and prolonged incubation with 50 mM 3-methylcrotonic yields a fully split spectrum (red). (F) Incubation of *Ta*Fdc with pentenoic (green) and hexenoic (orange) acids leads to a similar splitting in spectrum with both acids. (G) Incubation of *Ta*FdcV with pentenoic acid (green) immediately splits the spectrum whereas prolonged incubation with hexenoic acid (orange) does not induce full splitting. (H) Decay in *Ta*Fdc decarboxylase acitivity when exposed to light (orange) with a half-life of about 1 hour, compared to no changes in activity when stored in the dark. (I and J) Splitting in the UV-Vis spectra of *Ta*Fdc and *Ta*FdcV to peaks at 365 nm and 425 nm when irradiated with a 405 nm LED lamp (ThorLabs M405L3).

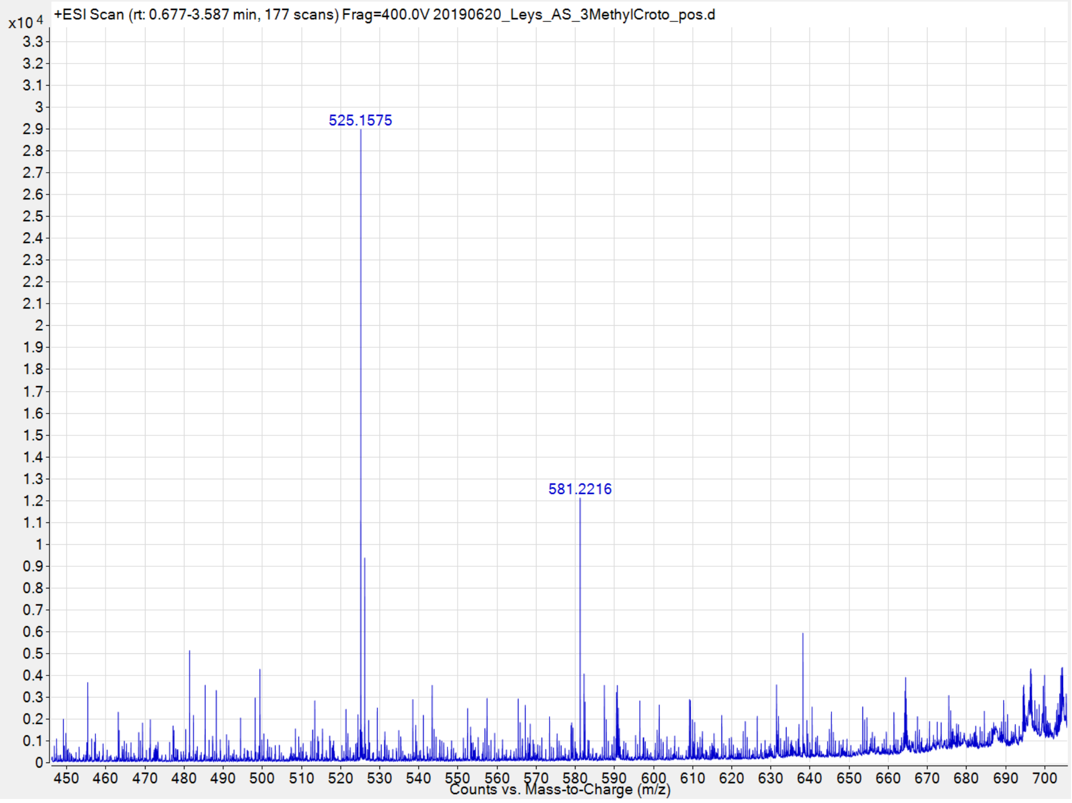

Supplementary Figure 3. ESI-MS spectrum of *Ta*FdcV incubated with 3-methylcrotonic acid followed by desalting showing the mass for prFMN^{iminium} and for the putative Int3 cycloadduct.

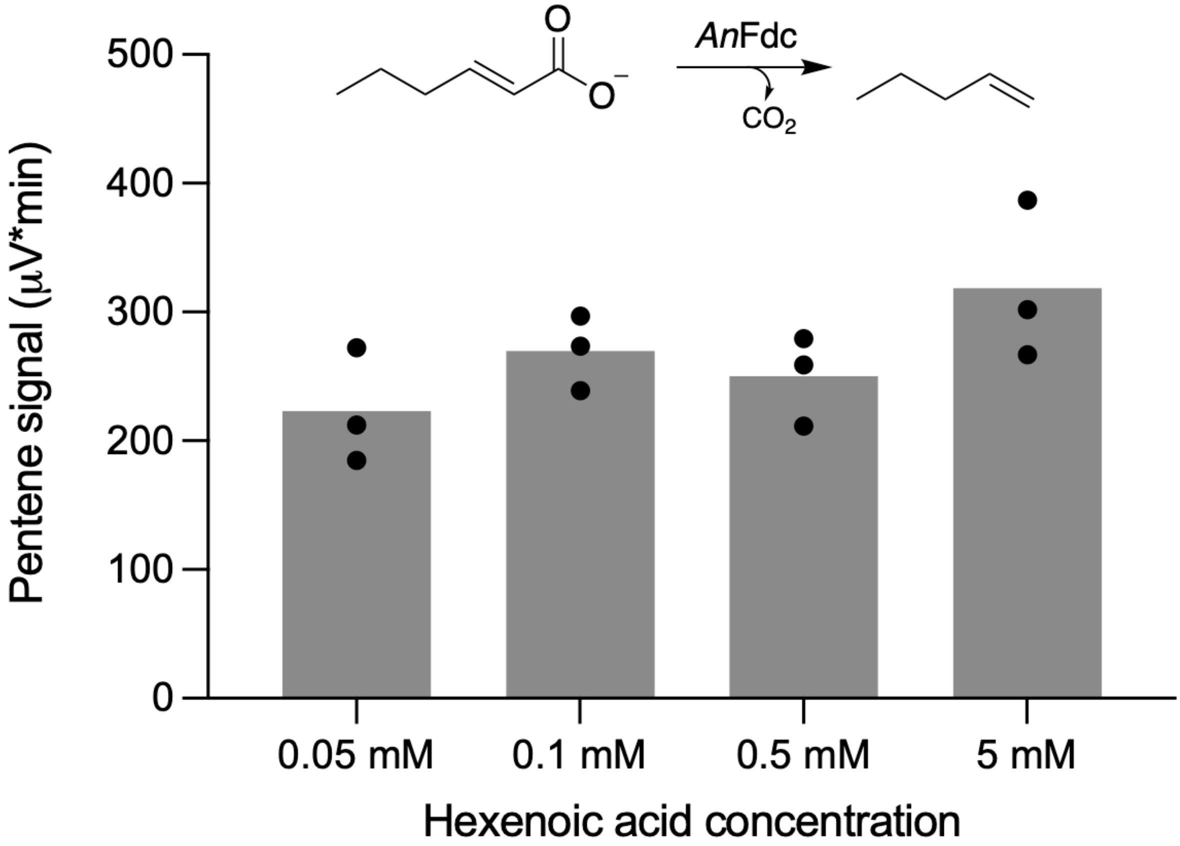

Supplementary Figure 4. *An*Fdc assay with hexenoic acid suggesting single turnover. Pentene detected by GC from manual headspace injection. 0.05 mM of *An*Fdc incubated with 0.05 mM to 5 mM hexenoic acid overnight at 18 °C, 180 rpm. Reaction buffer: 50 mM KCl, 50 mM NaPi, pH 6. Source data are provided as a Source Data file.

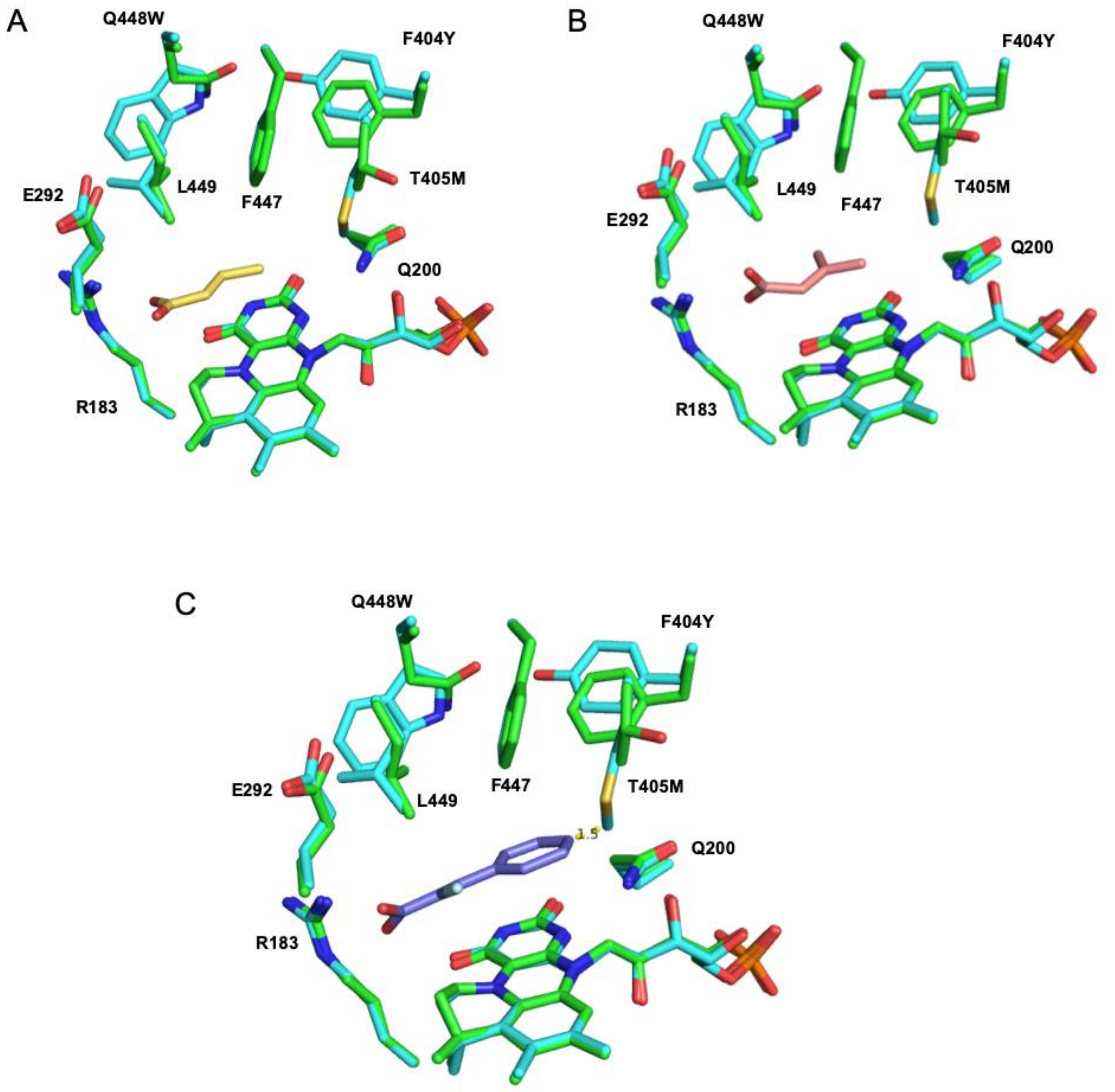

Supplementary Figure 5. Substrate binding at the active sites of *Ta*Fdc and *Ta*FdcV. Crotonic (A) and 3-methylcrotonic (B) acid binding at the active site of *Ta*FdcV (blue), as modelled with Vina docking, overlaid with *Ta*Fdc wild-type (green). (C) *Ta*FdcV (blue) and *Ta*Fdc wild-type (green) overlaid with *An*Fdc wild-type in complex with alpha-fluorocinnamic acid (PDB: 4ZAB) demonstrating the clash between the M405 residue and the phenyl ring.

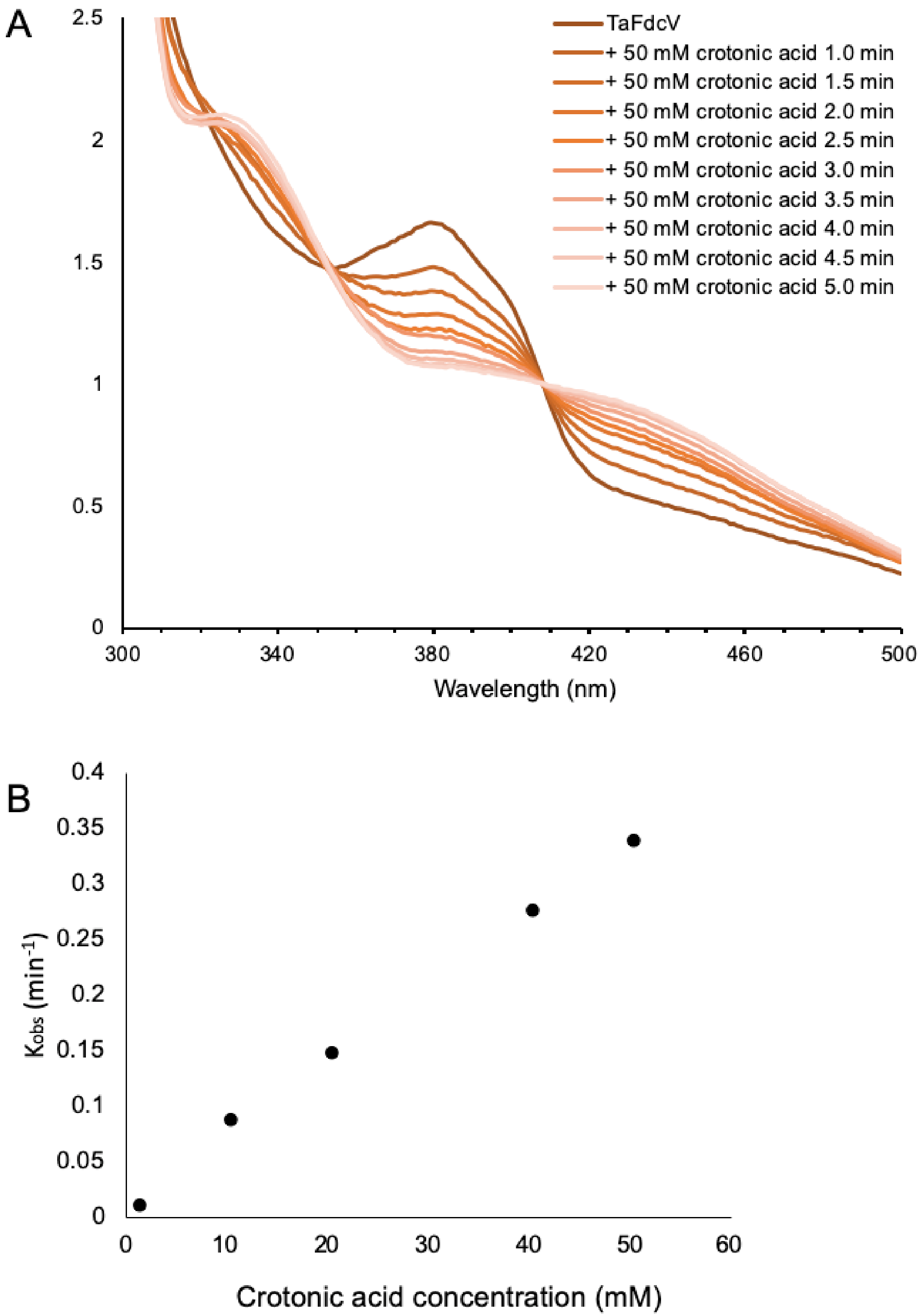

Supplementary Figure 6. *Ta*FdcV kinetics with crotonic acid. (A) Gradual split of the 380 nm prFMN peak upon addition of crotonic acid. (B) Observed rate of cycloadduct formation based on decrease in the 380 nm peak showing a linear relationship. Rate measured with 1 mM, 10 mM, 20 mM, 40 mM and 50 mM crotonic acid.

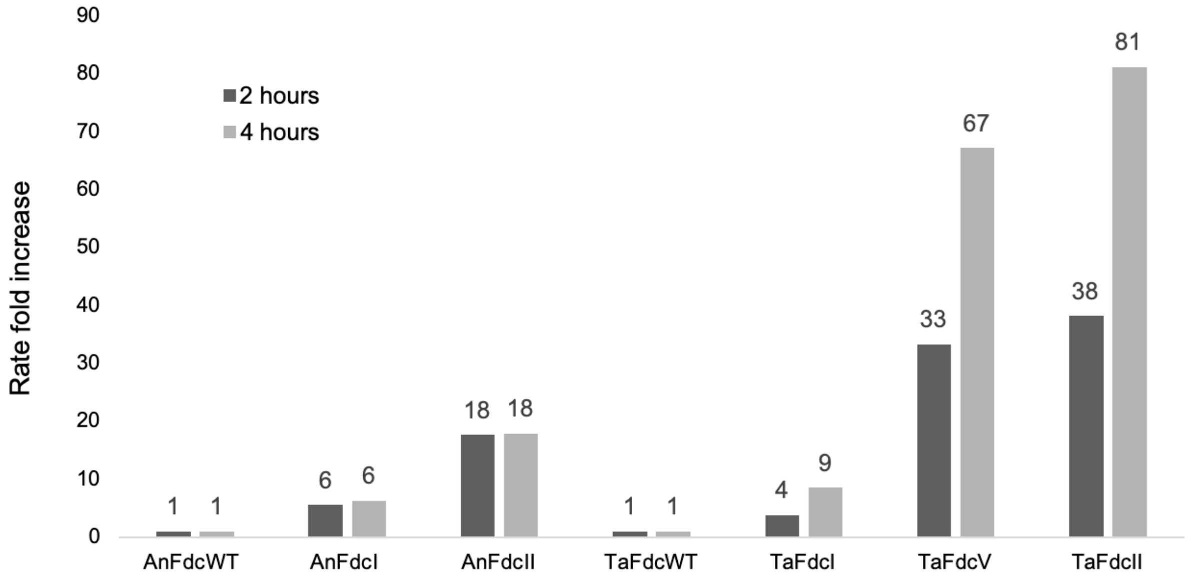

Supplementary Figure 7. Isobutene titer fold increase of *An*Fdc and *Ta*Fdc variants in relation to activity of corresponding wild-type enzyme, respectively. *In vitro* isobutene production by purified enzyme measured by GC after 2 hours (dark grey) and 4 hours (light grey). Source data are provided as a Source Data file.

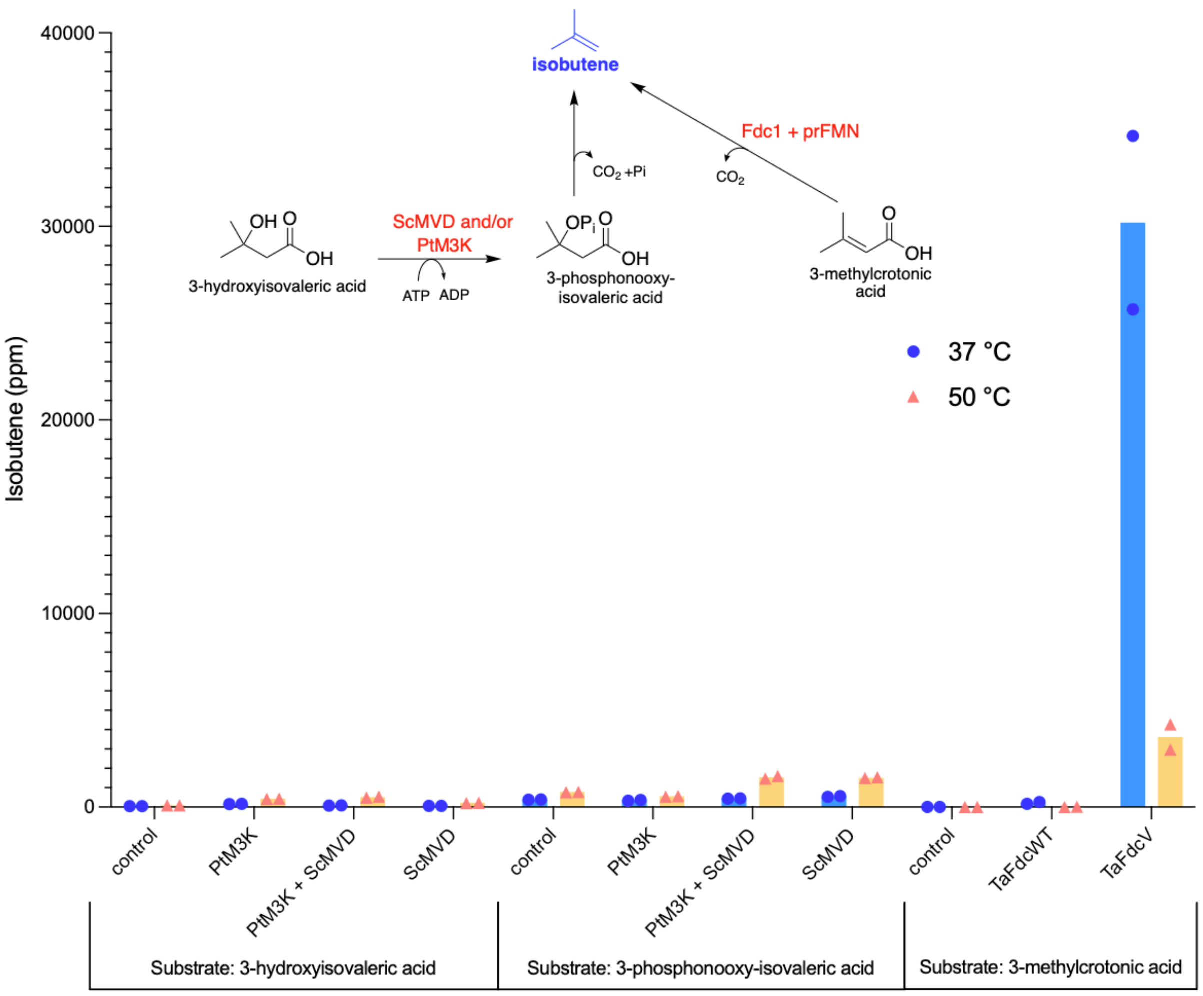

Supplementary Figure 8. Comparison of isobutene *in vitro* production levels, using *E. coli* cell lysate. An equal amount of lysate obtained from cells expressing respectively *Picrophilus torridus* mevalonate 3-kinase (PtM3K), *Saccharomyces cerevisiae* mevalonate diphosphate decarboxylase (ScMVD) or *Ta*Fdc wild-type/V (in combination with UbiX) was incubated with 50mM of respectively 3-hydroxyisovalerate/ATP, 3-phosphonooxy-isovalerate/ADP or 3-methylcrotonate. Following 4 h incubation at 37 °C (blue circles) and 50 °C (orange triangles), the isobutene content of the gas phase was analysed using GC. Under these conditions, *Ta*FdcV mediated isobutene levels exceeded the highest *Pt*M3K/*Sc*MVD levels by ~50-fold at 37 °C. The highest *Ta*FdcV isobutene conversion was approximately 11.5% of the substrate provided. Source data are provided as a Source Data file.

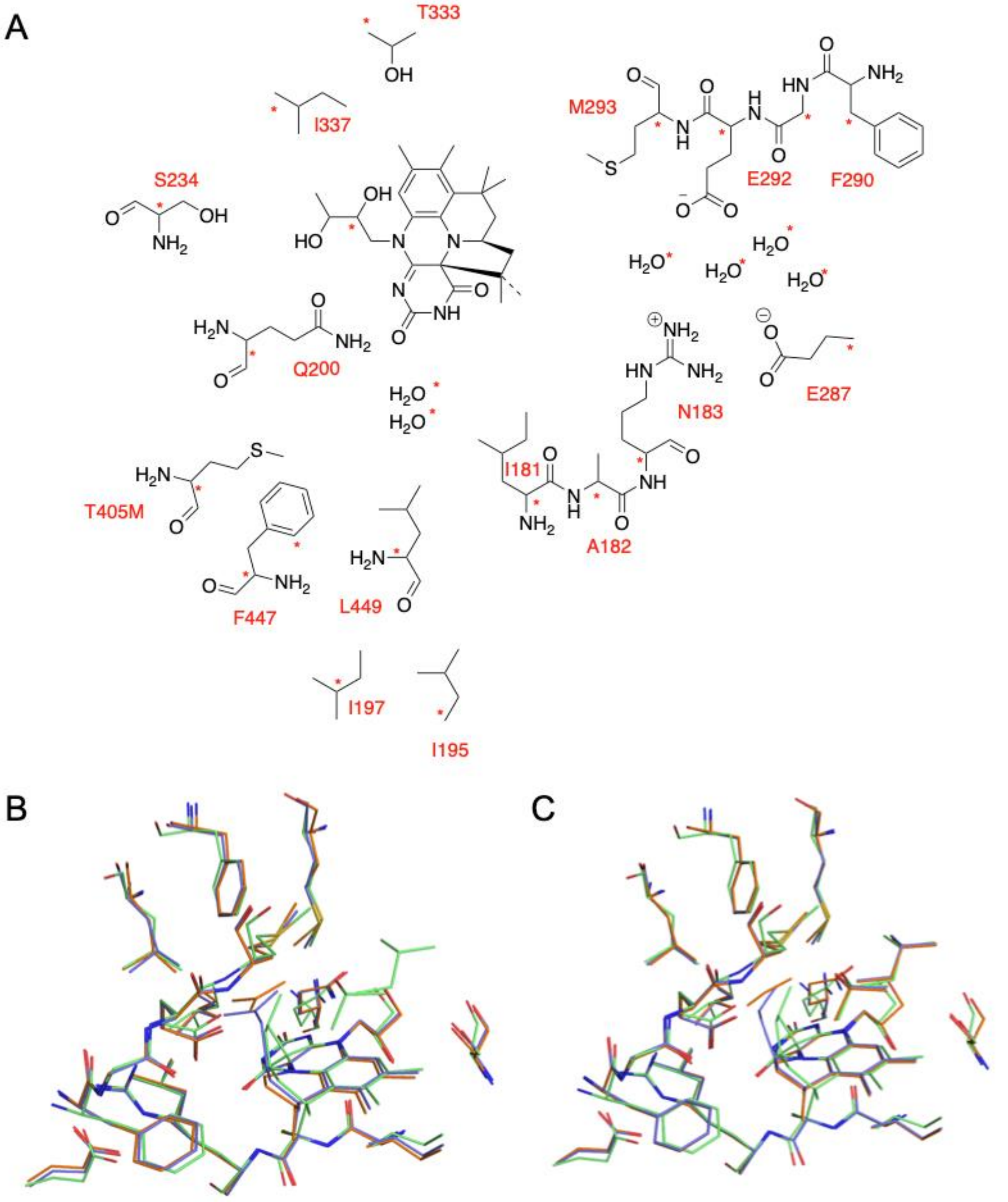

Supplementary Figure 9. DFT calculations. (A) Model of the active site of *Ta*FdcV used for DFT calculations based on the crystal structure of *Ta*FdcV co-crystallised with crotonic acid (7NF2) in **Int3**. Red asterisks denote fixed atoms. (B) Overlay of *Ta*FdcV crystal structure with crotonic acid adduct (green) and DFT optimized models of 3-methylcrotonic acid transition state (purple) and cycloelimination product isobutene (orange). (C) Overlay of the *Ta*FdcV crystal structure with crotonic acid adduct (green) and DFT optimized models of crotonic acid transition state (purple) and cycloelimination product propene (orange).
